# Dataset normalization for low carbon cities in a multi-criteria evaluation model

**DOI:** 10.1016/j.dib.2018.03.130

**Published:** 2018-03-31

**Authors:** Hossny Azizalrahman, Valid Hasyimi

**Affiliations:** Department of Urban and Regional Planning, King Abdulaziz University, Jeddah, Saudi Arabia

**Keywords:** Data normalization, Low carbon city, Scoring, Multi-criteria evaluation, Carbon emissions

## Abstract

Data in this article are related to a paper entitled “Towards a Generic Multi-criteria Evaluation Model for Low Carbon Cities”. This paper sets out a framework for data normalization in a multi-criteria evaluation model that was tested and validated in 15 cities. Data deals with measurable indicators such as GDP per capita, CO_2_ emissions per capita and public buses per capita. In addition to published papers, selected World Bank and Siemens reports were useful to operationalize and identify low carbon cities.

**Specifications Table**

**Table:** 

Subject area	*Engineering*
More specific subject area	*Low Carbon City, Multi-criteria Evaluation Modelling*
Type of data	*Data Files in Excel Format and Links*
How data was acquired	*Publicly available data sources online*
Data format	*Raw, analyzed*
Experimental factors	*Brief description of any pretreatment of samples*
Experimental features	*Very brief experimental description*
Data source location	**Pilot Cities**: *Stockholm, Vancouver, London, New York, Johannesburg, Beijing, Sydney, São Paulo, Mexico City and Tokyo.*
	**Tested Cities**: *Copenhagen, Bogota, New Delhi, Singapore and Seoul*
Data accessibility	*Data is available online on:*
	1. *World Bank* 2. *Air Plume* 3. *Siemens:*
	* Europe*
	* *https://www.siemens.com/entry/cc/features/greencityindex_international/all/en/pdf/report_en.pdf
	* Asia*
	* *http://sg.siemens.com/city_of_the_future/_docs/Asian-Green-City-Index.pdf
	* America*
	* *http://www.siemens.com/entry/cc/features/greencityindex_international/all/en/pdf/report_northamerica_en.pdf
	* Australia*
	* *http://www.siemens.com/entry/cc/features/greencityindex_international/all/en/pdf/report_latam_en.pdf
	4. *Official Website of every city*

**Value of the data**

• Data herein presents seven low-carbon cities which were determined after an evaluation of 15 selected cities.
• Normalization of raw data was fraught with difficulties due to limitation of data, data incompatibility, differences in scales/units and time frame, but was handed in the evaluation model.
• Low-carbon is a strong indication of sustainable cities but requires accurate and up-to-date data.
• Data that has been processed here could be easily utilized by other researchers and cities which attempt to embark on sustainability studies.

## 1 Data

The data of pilot and tested cities in this article [1] are derived from credible organizations such the World and Siemens and the official websites of selected cities. The data consists of:

**Table:** 

• GDP Per capita	(USD/capita)
• Proportion of tertiary industry to GDP	(%)
• Carbon productivity	(USD/ton)
• Proportion of renewable energy	(%)
• Energy intensity	(Mega Joules/USD)
• Proportion of public green space	(%)
• Population density	(People/km^2^)
• CO2 emission per capita	(ton/person)
• NO2 emission per capita	(μg/m^3^)
• Sulphur	(μg/m^3^)
• Suspended materials	(μg/m^3^)
• Public buses per capita	(buses/million person)
• Rail length per capita	(km/million person)
• Cars per capita	(cars/person)
• Solid waste generation per capita	(kg/capita/day)
• Share of waste collected and adequately disposed	(%)
• Share of waste to energy	(%)
• Share of material recycling	(%)
• Share of wastewater treated	(%)
• Water consumption intensity	(liter/person/day)

## 2 Experimental design, materials and methods

**Fig. 1:**
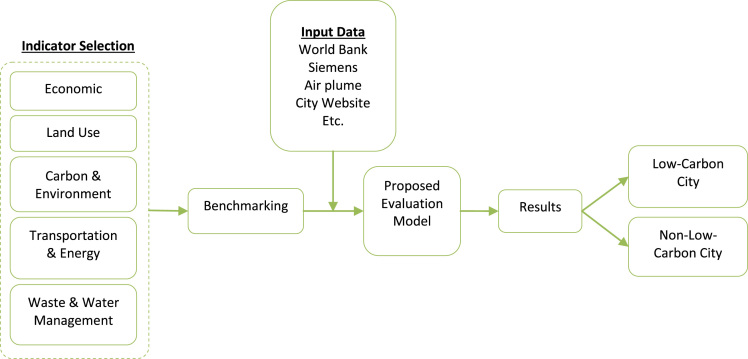
Data analysis framework.

The initial step of this research was to adjust entropy weight model by adding certain criteria weight to each criterion to the pilot cities [2]. The data analysis framework encompasses indicator selection, data input, benchmarking and evaluation model all leading to low carbon city identification (Fig. 1).

### 2.1 Modified entropy weight model

**Fig. 2:**
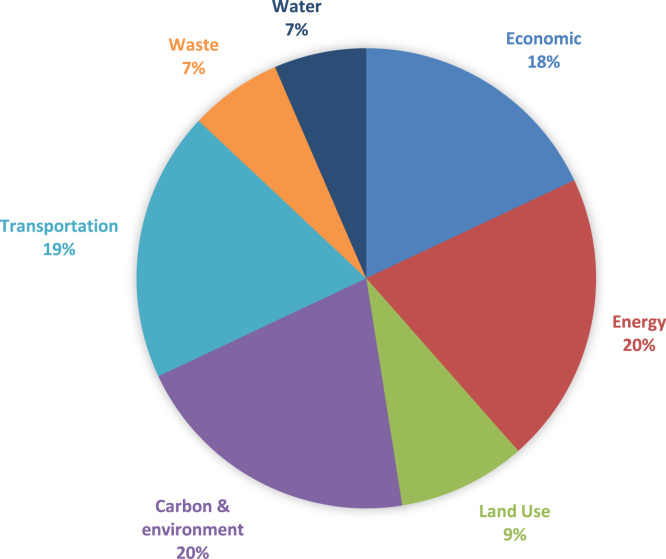
Relative criteria weight. Adapted from International Energy Agency, 2012; EPA, 2015; WDI, 2015; IPCC, 2006; and Global Atmospheric Research, 2000.

 The initial step of this research was to adjust entropy weight model by adding certain criteria weight to each criterion to the pilot cities [1]. Data in the entropy weight model has been modified by adding relative weight, the result of which can be seen in Fig. 2.

### 2.2 Proposed multi-criteria evaluation model for low carbon city

Detailed data of indicators for each city were obtained from [3]. Next step was to input data to the table of the proposed model after data normalization *has been made* using Eqs. (1), (2).

(1) $$y_{i}=\frac{x_{i}\;-\;x_{b}}{x_{b}}$$

(2) $$y_{i}=\frac{x_{b}\;-\;x_{i}}{x_{b}}$$

where $y_{i}$ is normalized data of assessed object on i indicator, $x_{i}$ is original value of the object on $i^{th}$ indicator, $x_{b}$ is benchmark value of $i^{th}$ indicator. While Eq. (1) is used for indicators with positive effects on carbon emissions level, Eq. (2) is used for indicators with negative effects [4].

The calculation of proposed evaluation model is shown in Eq. (3).

(3) $$S_{t}=\sum_{c=1}(S_{c}\times w_{c})$$

where $S_{t}$ is the total score of assessed city, $w_{c}$ is the weight factor of *c* category, and $S_{c}$ is total score of $y_{\mathrm{ic}}$ in $c^{th}$ category.

The calculation result of proposed entropy weight model and proposed multi-criteria evaluation model for low carbon city can be seen in Table 1.

**Table 1:** Comparison of results between modified entropy weight model and proposed multi-criteria evaluation model.

### 2.3 Tested data

**Fig. 3:**
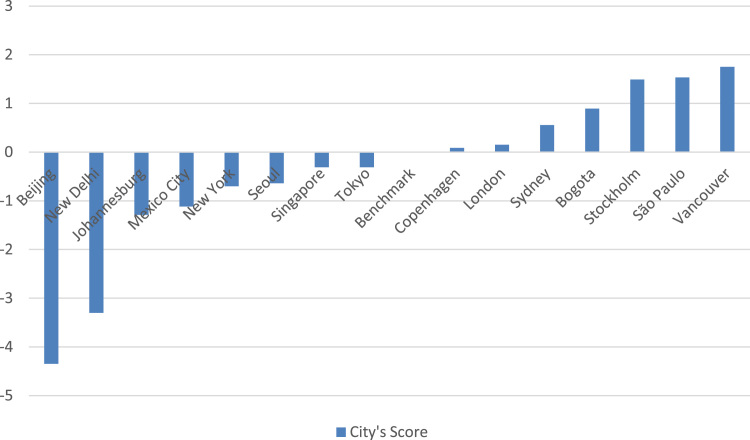
Low carbon city's score of 15 selected cities.

Data for the cities of Copenhagen, Bogotá, New Delhi, Singapore and Seoul were normalized to test model’s reliability and applicability. The 15 selected cities are charted in Fig. 3; those which score over the benchmark are low carbon cities and those which fall behind are not.

**Fig. 4:**
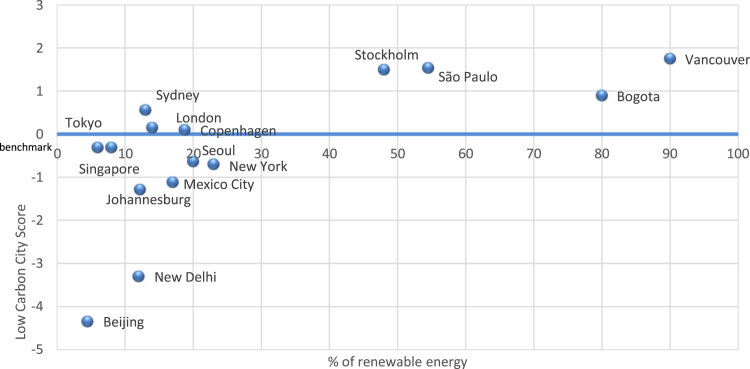
Correlation between renewable energy and sustainability score for selected cities.

### 2.4 Correlation between proportion of renewable energy and low carbon city's score for selected cities

Additional analysis of data to ascertain sustainability was made by correlating renewable energy and low carbon in selected cities as shown in Fig. 4. We can conclude that the proportion of renewable energy has strong positive correlation with low carbon city score. Cities which surpass the benchmark and are considered low carbon cities are: Sydney, London, Copenhagen, Stockholm, Sao Paulo, Bogota and Vancouver.
