# Supplementary figures and images for: Dataset normalization for low carbon cities in a multi-criteria evaluation model
Source: Data Brief. 2018 Mar 31;18:1111–6. doi: 10.1016/j.dib.2018.03.130 (PMC5996616; doi:10.1016/j.dib.2018.03.130)

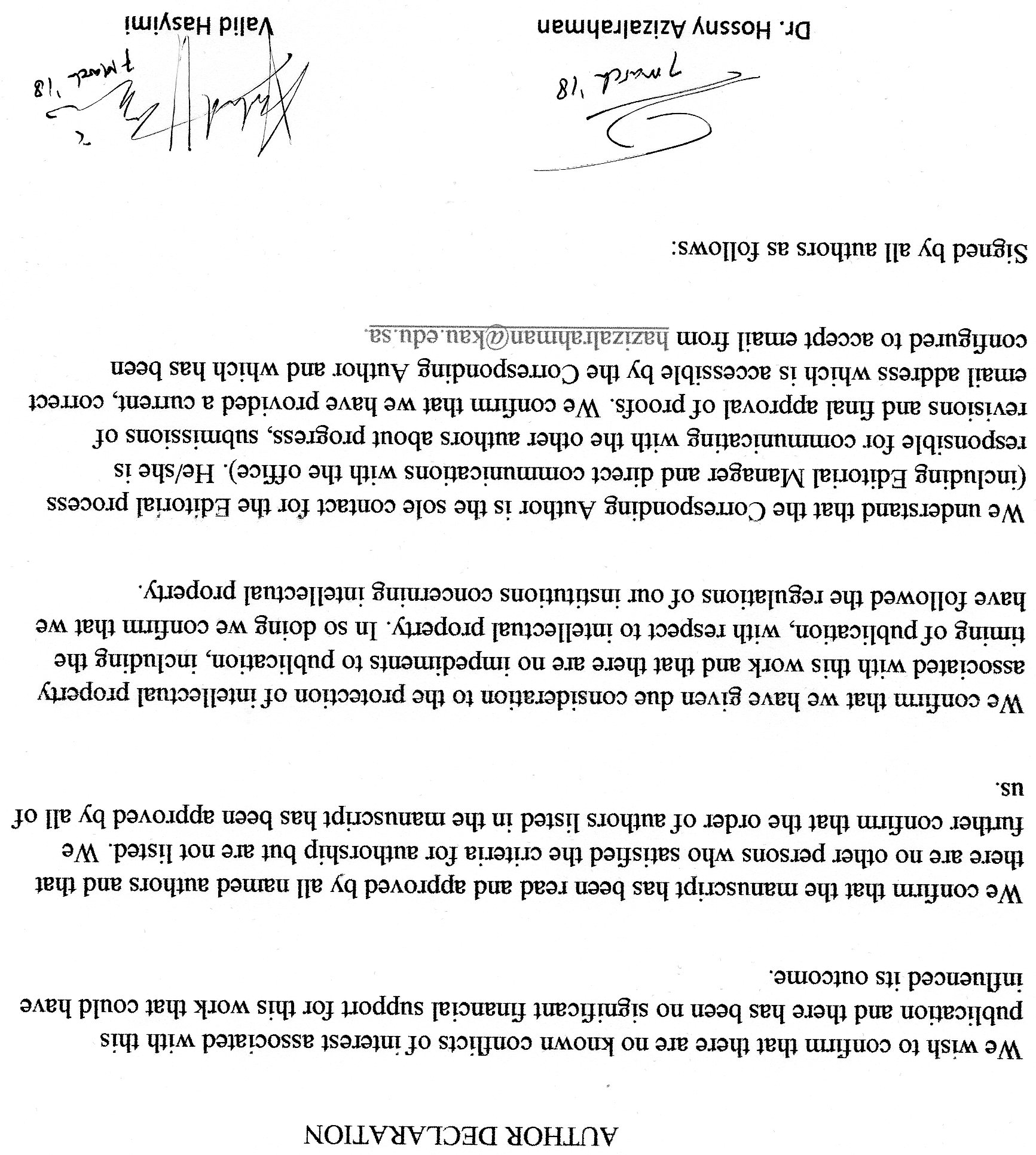

Supplement: Supplementary file 1 — Supplementary material [file mmc1.docx]
